# HTLV-I and –II seroprevalence among United States blood donors, 2000-2009

**DOI:** 10.1186/1742-4690-8-S1-A74

**Published:** 2011-06-06

**Authors:** Zhanna Kaidarova, Edward L Murphy

**Affiliations:** 1 Blood Systems Research Institute, San Francisco, CA 94118, USA; 2 University of California San Francisco, CA 94118, USA

## Background

HTLV-I and –II (HTLV-I/II) infection is prevalent at low levels in the United States, but recent reports on HTLV-I/II prevalence among blood donors are lacking.

## Methods

Computerized data on all first-time blood donors in a large network of United States blood centers was examined during the period 2000-2009. HTLV-I/II antibody was measured by enzyme immunoassay (EIA), with supplemental testing of all reactive samples using an EIA from an alternate manufacturer. Prevalence rates were calculated, and odds ratios (OR) and 95% confidence intervals (CI) for associations with demographic characteristics were assessed using multivariate logistic regression.

## Results

Among 1,904,155 first-time blood donors, HTLV-I/II prevalence decreased from 10 per 10,000 in 2000 to 5 per 10,000 in 2009 (p trend < 0.0001). Prevalence increased with age through middle age and then decreased in older age. HTLV-I/II infection was associated with female sex (OR =1.54, 95% CI 1.28-1.85), age 50-59 (OR=10.15, 95% CI 6.16-16.72), Black (OR=5.69, 95% CI 4.38-7.38) and Hispanic (OR=2.59, 95% CI 2.06-3.26) race/ethnicity, and inversely with university education (OR=0.59, 95% CI 0.45-0.76). There was minimal geographic variation, although donors from the southwestern US had slightly higher prevalence.

## Conclusions

HTLV-I/II prevalence is decreasing slowly among US blood donors, consistent with a birth cohort effect whereby donors born in the 1950s and 60s had higher prevalence rates compared to younger donors. HTLV-I/II associations with female sex, non-white race and lower educational achievement are consistent with previous studies. With 3.2 million first-time donors annually nationwide, US blood banks detect several hundred HTLV-I/II infections per year.

